# Recent lake expansion triggered the adaptive radiation of freshwater snails in the ancient Lake Biwa

**DOI:** 10.1002/evl3.92

**Published:** 2018-11-30

**Authors:** Osamu Miura, Misako Urabe, Tomohiro Nishimura, Katsuki Nakai, Satoshi Chiba

**Affiliations:** ^1^ Faculty of Agriculture and Marine Science Kochi University 200 Monobe Nankoku Kochi 783‐8502 Japan; ^2^ Department of Ecosystem Studies, School of Environmental Science The University of Shiga Prefecture 2500 Hassaka‐cho Hikone, Shiga 522‐8533 Japan; ^3^ Current address: Cawthron Institute 98 Halifax Street East Nelson 7010 New Zealand; ^4^ Lake Biwa Museum 1091 Oroshimo Kusatsu Shiga 525‐0001 Japan; ^5^ Department of Environmental Life Sciences, Graduate School of Life Sciences Tohoku University Kawauchi 41 Aoba‐ku Sendai 980‐0862 Japan

**Keywords:** ancient lakes, adaptive radiation, lake‐size changes, fossils, genome DNA analysis

## Abstract

Lake expansion that leads to the formation of new habitats has potential to drive intralacustrine diversification. The ancient Lake Biwa in central Japan has historically experienced substantial changes in the lake size, and it provides a useful system for evaluating the role of lake‐size fluctuations in the diversification of endemic fauna. Here, we used genome‐wide DNA analyses and reconstructed the diversification history of the endemic freshwater snails belonging to the subgenus *Biwamelania* with respect to the geological history of Lake Biwa. We found that two genetically distinct snail lineages independently colonized Lake Biwa and they concurrently and rapidly radiated into 15 extant *Biwamelania* species. A combination of paleontological evidence and molecular dating technique demonstrated that the radiation of *Biwamelania* was tightly linked to the latest enlargement of the lake about 0.4 million years ago and suggested that increased ecological opportunity associated with the lake expansion drove the rapid adaptive radiation. We propose that the *Biwamelania* snails in Lake Biwa offer a promising new system for understanding the association between the geological history of the lake and rapid intralacustrine diversification.

**Impact summary**

Ancient lakes often harbor highly diverse faunas with many endemics and contributed to advance our understanding of intralacustrine diversification. More than hundreds of cichlid species were rapidly evolved in the Great East African Lakes and there is also a great diversity of amphipods that evolved in the Siberian Lake Baikal. Here, we introduce a new system for further developing the model of endemic diversification in ancient lakes. The Japanese ancient Lake Biwa harbors a suite of endemic freshwater snails in the subgenus *Biwamelania*. Using a combination of genome DNA analyses and paleontological evidence, we found that these *Biwamelania* species rapidly radiated with exploiting a variety of habitats in response to the latest expansion of the lake about 0.4 million years ago. The clear geology and abundant fossils in Lake Biwa can provide a framework for determining the evolutionary factors that have facilitated rapid species diversifications within ancient lakes.

Lake‐size changes are one of the major drivers of extinction and speciation in lake fauna (Sturmbauer and Meyer 1992; Sturmbauer 1998; Kornfield and Smith 2000; Sturmbauer et al. 2001; Sturmbauer et al. 2011). A shallowing and reduction of lake area will elevate the extinction rate because of intense competition in the shrinking environments. In contrast, a deepening and enlargement of lake will promote population expansion and increase the speciation rate because of colonization to new habitats, reduced competition, and population subdivisions (Sturmbauer 1998). For example, the cichlids in the Great East African Lakes have shown that intralacustrine radiations occur upon lake refilling after severe drought (Owen et al. 1990; Sturmbauer and Meyer 1992; Sturmbauer 1998; Kornfield and Smith 2000; Sturmbauer et al. 2001; Genner et al. 2010; Sturmbauer et al. 2011). Severe drought could have extinguished many cichlid species while survived cichlids could have encountered several empty niches upon the lake expansion, which are considered to partly facilitate the rapid adaptive radiation in the African cichlids (Sturmbauer et al. 2011).

The cichlids in the Great East African Lakes have caused a major development in models for adaptive radiation (Albertson et al. 1999; Sturmbauer et al. 2001; Kocher 2004; Seehausen 2006; Sturmbauer et al. 2011). While these species flocks exhibit intralacustrine radiations and will certainly help in obtaining more information on evolutionary dynamics of species diversification, the intralacustrine radiations of endemic invertebrates such as mollusks may also have a great potential in contributing to our knowledge on species diversification (von Rintelen et al. 2004; Albrecht et al. 2006; Albrecht et al. 2008; Glaubrecht 2008; Schultheiß et al. 2009; Van Bocxlaer and Hunt 2013; Van Bocxlaer 2017). Because mollusks are less mobile, they are likely to become geographically isolated to a higher degree. Further, because of their calcareous shells, mollusks have a high fossilization potential. These behavioral and structural characters of mollusks enable us to effectively access to the information on both recent and past species histories.

The subgenus *Biwamelania* (Mollusca: Caenogastropoda: Semisulcospiridae) is composed of 15 extant species and 10 fossil species (Matsuoka 1987; Nishino and Watanabe 2000; Matsuoka and Miura 2018) and it is the most diverse endemic group in Lake Biwa (Watanabe and Nishino 1995; Nishino and Watanabe 2000; Kihira et al. 2003). Lake Biwa is an ancient lake located in central Honshu in Japan (Fig. 1A). The lake has experienced substantial lake‐size changes throughout its history (Satoguchi 2012) (see Fig. 1B). First, the relatively shallow and small lake was formed south of the current Lake Biwa about four million years ago, and the lake depth and area substantially increased between 3.2 and 2.6 million years ago. After this deep and large lake period, the lake eventually moved north and almost dried up (but remained as swamps) until 1.8 million years ago. The lake basin moved to the location near the current Lake Biwa and was refilled about 1.2 million years ago. The enlargement of Lake Biwa to its present volume was estimated to have occurred approximately 0.4 million years ago (Satoguchi 2012). These lake‐size changes would have repeatedly affected lacustrine habitat conditions and should have influenced the distribution and diversity of the endemic snails (Matsuoka 1987).

**Figure 1:**
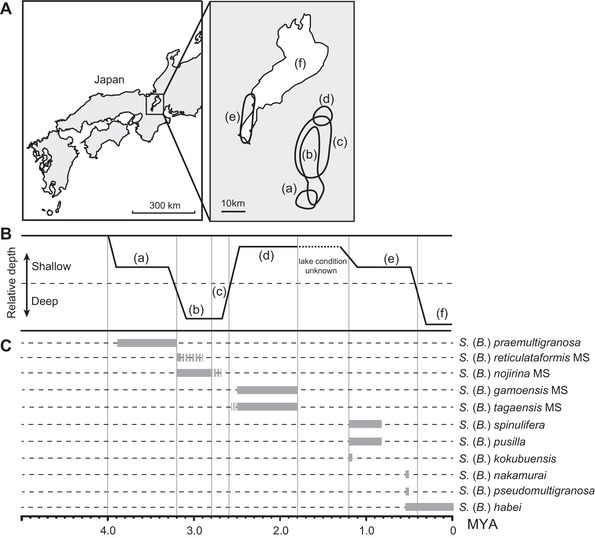
Histories of Lake Biwa and the *Biwamelania* snails. The geographical locations and area size of the past and current Lake Biwa (A). The history of Lake Biwa can be divided into six stages with respect to geographical location (a–f). The relative depth of the past and current Lake Biwa (B). The relative depth was inferred on the basis of geological information published by Kawabe (1994) and Satoguchi (2012). Alphabets represent the stage of the lake shown in (A). Fossils of *Biwamelania* species and their known time ranges (C). The ages of the fossils were referred from Matsuoka (1987) and were further corrected on the basis of a recent stratigraphic study by Satoguchi and Nagahashi (2012).

The fossil records have demonstrated that *Biwamelania* species experienced extinction and speciation events associated with the lake‐size changes (Fig. 1C). The oldest *Biwamelania* species, *Semisulcospira* (*Biwamelania*) *praemultigranosa*, first appeared at Paleo‐lake Tokai about 3.9 million years ago and it colonized Lake Biwa about 3.6 million years ago (Matsuoka, 1987, 2001); however, it became extinct during the deepening event of the lake about 3.2 million years ago. *Semisulcospira* (*Biwamelania*) *reticulataformis* MS and *Semisulcospira* (*Biwamelania*) *nojirina* MS subsequently emerged in the deep and large lake, but they had been disappeared before or during shallowing event of the lake about 2.6 million years ago. The shallow swamps between 2.6 and 1.8 million years ago harbored *Semisulcospira* (*Biwamelania*) *gamoensis* MS and *Semisulcospira* (*Biwamelania*) *tagaensis* MS, and these species were replaced by six *Biwamelania* species when the lake was refilled about 1.2 million years ago. Of the six species, five species were extirpated before the latest expansion of the lake about 0.4 million years ago (Matsuoka 1987; Matsuoka and Miura 2018).

Fifteen *Biwamelania* species are present in the current Lake Biwa, but most of them were not found from fossil records, except for *Semisulcospira* (*Biwamelania*) *habei* (Matsuoka 1987). This suggests that the most of extant *Biwamelania* species may have rapidly radiated after the latest expansion of the lake (Nishino and Watanabe 2000). However, this scenario should be carefully tested because the fossil record is generally less complete at lower taxonomic levels (Benton 1995). Although it has become possible to reconstruct the evolutionary history of a focal group on the basis of DNA variations in the extant species, previous molecular studies have demonstrated that the molecular phylogenies of the genus *Semisulcospira* on the basis of a single or few genes did not accurately reflect their evolutionary relationship because of the retention of ancestral polymorphisms or introgression (Lee et al. 2007; Miura et al. 2013; Köhler 2016). A previous study performed using several allozyme loci also failed to resolve their phylogenetic relationships because of insufficient variations in these loci (Kamiya et al. 2011). Only a method that assays variations in a large number of unlinked loci is likely to minimize the risk of inaccurate inference of phylogenetic relationships (Albertson et al. 1999; Carstens and Knowles 2007). In this study, we used a genome‐wide DNA analyses based on a double digest restriction site associated DNA library sequencing technique to infer the robust phylogenetic relationship of *Biwamelania* and related riverine species. We tested the hypothesis of recent radiation of the *Biwamelania* snails associated with the latest expansion of the lake and we further evaluated what ecological factors facilitated the radiation of the *Biwamelania* snails. Finally, we highlighted how this system can serve as a useful system to provide insight into what processes drive adaptive radiation in ancient lakes.

## Methods

### SAMPLE COLLECTIONS

Fifty‐four individuals of 14 endemic species in Lake Biwa were obtained from 15 sites in Lake Biwa and its drainage (see Table S1 for details, see also Fig. S1 for their shell morphology). We used snorkeling and SCUBA to collect the samples from deeper habitats and obtained *Semisulcospira* (*Biwamelania*) *multigranosa* and one specimen of *Semisulcospira* (*Biwamelania*) *reticulata* from a fisherman who dredged near Okishima Island in the lake. We could not find *Semisulcospira* (*Biwamelania*) *ourense* despite our sampling efforts. *Semisulcospira* (*Biwamelania*) *ourense* is an extremely rare species, and only a handful of individuals were recorded after its description (Watanabe and Nishino 1995; Kihira et al. 2003). Most of the *Biwamelania* species were collected from their type localities, although the type localities of *Semisulcospira* (*Biwamelania*) *decipiens*, *Semisulcospira* (*Biwamelania*) *niponica*, and *Semisulcospira* (*Biwamelania*) *multigranosa* are recorded just as “Lake Biwa”. In addition to endemic species in Lake Biwa, 42 individuals of the riverine species, *Semisulcospira libertina*, *Semisulcospira reiniana*, and *Semisulcospira kurodai* were collected from 29, four, and one sites, respectively. We identified these species based on adult shell features, and number and shape of embryos following the procedures of Davis (1969) and Watanabe and Nishino (1995). Snails were either fixed in 95% ethanol, stored at –30°C or both for molecular analyses. We isolated genome DNA using a modified CTAB procedure described by Miura et al. (2012). Some of these specimens were used in the former mtDNA study (Miura et al. 2013).

### GENOME DNA SEQUENCING AND IDENTIFICATION OF RAD LOCI

We used 104 genome DNA samples (Table S1) for a double digest restriction site associated DNA library (ddRAD) sequencing technique, as described by Peterson et al. (2012) with a slight modification. Briefly, the extracted DNA from each individual was further purified using a nucleospin gDNA clean‐up kit (Macherey‐Nagel) with addition of RNase A. Approximately 30 ng of DNA were digested using two restriction enzymes (*Eco*RI and *Msp*I). P1 and P2 adapters from Peterson et al. (2012) were ligated to the DNA fragments of each individual. The ligated samples were multiplexed and purified using a nucleospin gDNA clean‐up kit. An E‐gel size select agarose gel (Invitrogen, CA) was used to collect 300–350 bp DNA fragments. We amplified the DNA fragments in eight single PCR reactions. The PCR products were combined and cleaned using E‐gel size select agarose gel and the nucleospin gDNA clean‐up kit. The constructed DNA library was sent to Genome Quebec Innovation Center and sequenced using Illumina Hiseq 2000 single‐end sequencing, yielding maximum read lengths of 100 bp.

Raw sequence reads were processed using pyRAD 3.0.66 (Eaton 2014). Sequences were de‐multiplexed using their sample‐specific barcode without allowing any mismatches. The restriction site and barcode were removed from each sequence. A nucleotide base with a FASTQ quality score less than 20 was replaced with N. Sequences having more than 5% Ns were discarded. Sequences within each sample were clustered using VSEARCH (https://github.com/torognes/vsearch) with an 85% similarity threshold, following the pyRAD SE ddRAD tutorial. Within‐sample clusters with fewer than 10 sequences were excluded to ensure accurate base calls. Consensus sequences were created based on the clusters with consideration of the error rate and heterozygosity. Consensus sequences from all samples were clustered using the same similarity threshold that was applied in the within‐sample clustering. The resulting across‐sample clusters were aligned with MUSCLE (Edgar 2004). Any clusters having more than 5% shared polymorphic sites were discarded, because a shared heterozygous site across many samples likely represents clustering of paralogs (Hohenlohe et al. 2011). Clusters shared among fewer than 50 individuals were excluded, and the remaining clusters were treated as ddRAD loci (Table S1).

### PHYLOGENETIC ANALYSES AND DEMOGRAPHIC INFERENCES

The ddRAD sequences were concatenated into a single sequence alignment by an output function in pyRAD. Phylogenetic analysis was conducted by maximum likelihood (ML) algorism, using RAxML 8.0.20 (Stamatakis 2014) with general time reversible and gamma model. Node robustness was assessed using bootstrapping and 100 replicates. Other semisulcospirid species, *Semisulcospira extensa*, *Parajuga* sp., *Juga silicula*, and *Juga plicifera* were selected as outgroups, based on Lee et al. (2007), Strong and Köhler (2009), and Köhler (2016). As concatenation of genome DNA sequence data can be problematic because of spuriously high bootstrap supports for incorrect partitions (Gadagkar et al. 2005), we also inferred species trees based on multispecies coalescent model. Gene tree for each locus was estimated by RAxML with general time reversible and gamma model using the MAGNET pipeline (http://github.com/justincbagley/MAGNET). We then used ASTRAL III (Mirarab and Warnow 2015), which estimates a species tree that agrees with the largest number of quartet trees within a set of unrooted gene trees.

We estimated divergence time of the *Biwamelania* species using BPP 3.3 (Yang 2015). This analysis assumes no recombination among loci, neutral clock‐like evolution with JC69 mutation model, and no migration among species. Because the Bayesian analyses using the multispecies coalescent are computationally expensive, we used the subset of individuals and ddRAD loci shared among all those individuals to reduce the size of the dataset. We used species tree topology estimated by ASTRAL III and estimated relative divergence time (τ) and population size (θ) at each node. The prior for τ was Gamma (2, 250) and θ is Gamma (2, 1000). We converted τ value to actual time (t) using fossil calibration. The oldest fossil *Biwamelania* species, *S*. (*B*.) *praemultigranosa*, is recorded from the Ueno and Iga Formation of the Kobiwako Group and Kameyama Formation of the Tokai Group (3.9–3.2 million years ago) (Matsuoka, 1987, 2001). *Semisulcospira* (*Biwamelania*) *praemultigranosa* has an elongated conical shell outline and a small number (two or three) of basal cords on the body whorl, which correspond to diagnostic characters of the subgenus *Biwamelania* (Matsuoka 1985). This fossil species is the prospective earliest stem lineage of *Biwamelania*. Therefore, the age of the node representing the split of the basal group of *Biwamelania* [*S*. (*B*.) *habei* group] from the other Japanese *Semisulcospira* was set for 3.9 million years ago, which represents the first appearance of the subgenus *Biwamelania* (Matsuoka 2001) (see also Fig. 1). We used TRACER v. 1.6 and FIGTREE v. 1.4.2 (Drummond and Rambaut 2007) to check for convergence and to visualize the results. We also estimated net diversification rate of the *Biwamelania* and related riverine species using BAMM 2.5.0 (Rabosky 2014), based on the tree obtained by the BPP analysis. MCMC simulations in BAMM were run for 100 million steps, sampling parameters every 10,000 steps, and the MCMC results were analyzed within the R package BAMMtools (Rabosky et al. 2014).

The demographic histories of the *Biwamelania* species were reconstructed using Extended Bayesian Skyline Plot (EBSP) analysis (Heled and Drummond 2008), implemented in BEAST2 (Bouckaert et al. 2014). For each *Biwamelania* species, we randomly selected 200 loci that are shared by all individuals within the species. We used the same evolutionary model (JC69) as in the above BPP analysis and used the mutation rate estimated by the BPP analysis. The analyses were run for 1.5 billion generations, sampled every 50,000 steps and first 10–20% of samples were discarded as burnin. We used TRACER v. 1.6 to check convergence, and up to three independent runs were combined if the runs were not converged. We reduced the number of loci to 100 for *Semisulcospira* (*Biwamelania*) *nakasekoae* and *Semisulcospira* (*Biwamelania*) *takeshimensis* because their parameters did not converge in a reasonable computation time. The estimated relative population size was plotted against time for each *Biwamelania* species using R v3.3 (https://www.r-project.org).

### ECOLOGICAL ASSESSMENTS

To evaluate habitat usage patterns of the *Biwamelania* species, we classified substrate types of the sampling locations into the six categories: mud, sandy mud, sand, sandy gravel, pebble, and rock. Sandy mud is a mixture of mud and sand, and sandy gravel is a mixture of sand and gravel. We roughly identified the substrate types by eye at the field based on dominant particle size. We also recorded the depth range at the sampling locations. We then integrated our dataset and the habitat information of each *Biwamelania* species reported in the study of Watanabe and Nishino (1995), which contain about a hundred sampling points in Lake Biwa. Note that the numbers of the sampling locations for some species are limited as these species are distributed in restricted locations (see Fig. S2). We used chi‐square test to evaluate difference in habitat types among *Biwamelania* species. We used mid‐range as a representative depth at each sampling location and evaluated the difference in habitat depth among *Biwamelania* species. We compared the distribution of *Biwamelania* species along the lake depth using a general linear model. These statistical tests were performed using JMP V. 9.0 (SAS Institute).

## Results

We obtained 0.4 to 15.3 million reads for each individual after de‐multiplexing the Illumina Hiseq raw dataset (Table S1). The average number of clusters greater than nine sequences was 15,271.1; the average coverage achieved per individual per loci was 55.7 (Table S1). There were 6,285 loci with a total alignment length of 597,943 bp and the dataset had 31% of missing data.

**Figure 2:**
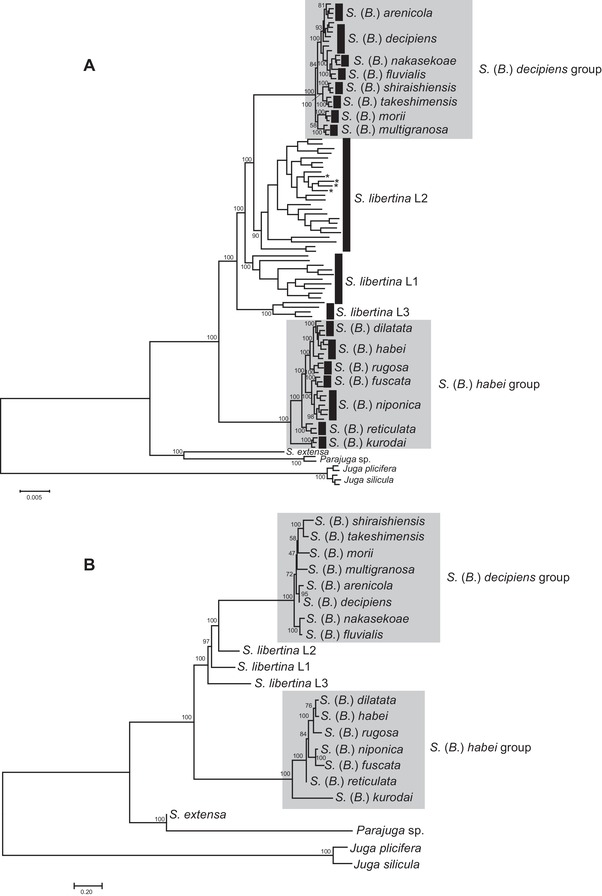
Maximum likelihood tree based on the concatenated ddRAD loci (A) and species tree based on the multispecies coalescent model (B). Asterisks at the terminal node in the concatenated ML tree indicate *S. reiniana*. Numbers near major nodes are the support values. The scale bar represents the mean number of nucleotide substitutions per site for the ML tree, and coalescent units for the species tree.

**Figure 3:**
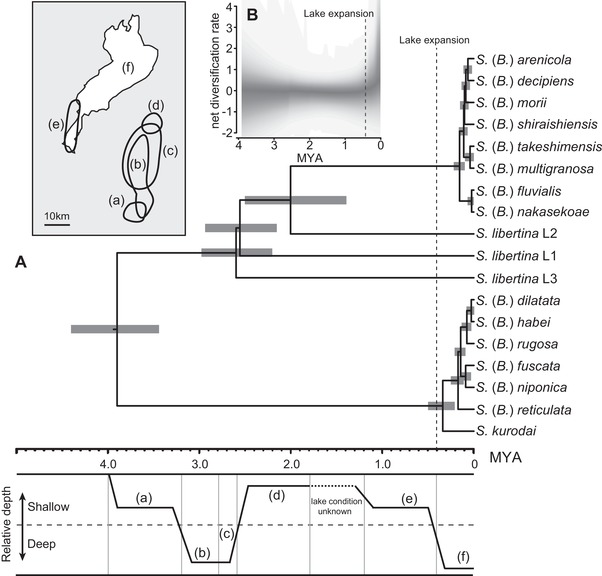
Phylogenetic evidence for the recent radiation of the subgenus *Biwamelania* (A). Divergence times were estimated for the *Biwamelania* species and related riverine species, under the multispecies coalescent model. Horizontal bars represent the upper and lower interval bounds for 95% of the highest posterior densities (HPDs). The geographical locations and relative depth of the past and current Lake Biwa are also shown (see details in Fig. 1). Plot of net diversification rate through time based on BAMM analysis (B). Shaded areas denote 90% Bayesian credibility intervals.

**Figure 4:**
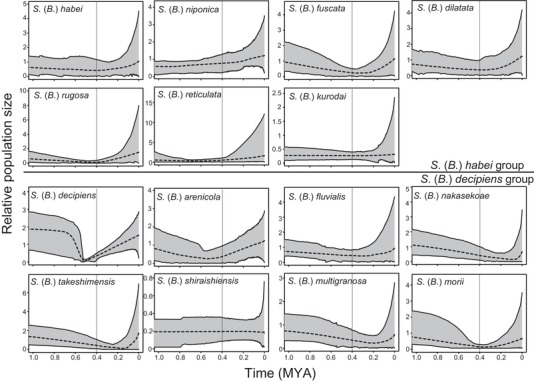
The results of Extended Bayesian Skyline Plots for the *Biwamelania* snails. The dotted line represents the median value for the relative population size, and the grey area indicates the 95% upper and lower credible limits. The bars at 0.4 million years ago indicate the time for the latest expansion of Lake Biwa.

The ML tree based on the concatenated ddRAD sequences is shown in Fig. 2A. There were five genetically well‐supported clades detected in *Semisulcospira* spp. All of these clades were supported by the highest bootstrap value (100%). *Semisulcospira libertina* L1 was distributed at the east side of Japan while *S. libertina* L2 was distributed at the west side of Japan. *Semisulcospira reiniana* was included in *S. libertina* L2. *Semisulcospira libertina* L3 was exclusively observed at the sites in Korea. The clear monophyly and a high level of divergence among the clades L1–3 suggest that these clades represent distinct biological species. However, we do not assign scientific names to these clades because the taxonomic revision is not the goal of this study. There were two largely separated clades in the Lake Biwa endemic species (Fig. 2A). One clade at basal position included *Semisulcospira* (*Biwamelania*) *habei*, *Semisulcospira* (*Biwamelania*) *dilatata*, *Semisulcospira* (*Biwamelania*) *rugosa*, *Semisulcospira* (*Biwamelania*) *fuscata*, *Semisulcospira* (*Biwamelania*) *niponica*, and *Semisulcospira* (*Biwamelania*) *reticulata*; this clade also included non‐endemic species, *Semisulcospira kurodai*. The other clade included *Semisulcospira* (*Biwamelania*) *decipiens*, *Semisulcospira* (*Biwamelania*) *nakasekoae*, *Semisulcospira* (*Biwamelania*) *fluvialis*, *Semisulcospira* (*Biwamelania*) *arenicola*, *Semisulcospira* (*Biwamelania*) *takeshimensis*, *Semisulcospira* (*Biwamelania*) *shiraishiensis*, *Semisulcospira* (*Biwamelania*) *multigranosa*, and *Semisulcospira* (*Biwamelania*) *morii*. We followed the reports by Kamiya et al. (2011) by calling the former clade as the *S*. (*B*.) *habei* group and the later clade as the *S*. (*B*.) *decipiens* group. The species tree based on multispecies coalescent models was similar to that of the concatenated ML tree (Fig. 2B). However, the species tree showed that the phylogenetic positions of the species within the *S*. (*B*.) *decipiens* groups were often uncertain. The ddRAD loci shared among all selected individuals (in total of 403 loci) were used for the divergence time estimates. The estimated divergence times for major clades and the Lake Biwa endemics are shown in Fig. 3A. The estimated mutation rate of *Biwamelania* was 1.06 ×10^−9^ substitution per year, which is comparable to the mutation rate in other organisms in diverse taxonomic group (Lynch 2010). The BAMM analysis demonstrated that the net diversification rate was increased after the latest enlargement of Lake Biwa (Fig. 3B). The EBSP analyses further exhibited that the most of *Biwamelania* species have experienced population expansions during and after the latest enlargement of the lake (Fig. 4).

**Figure 5:**
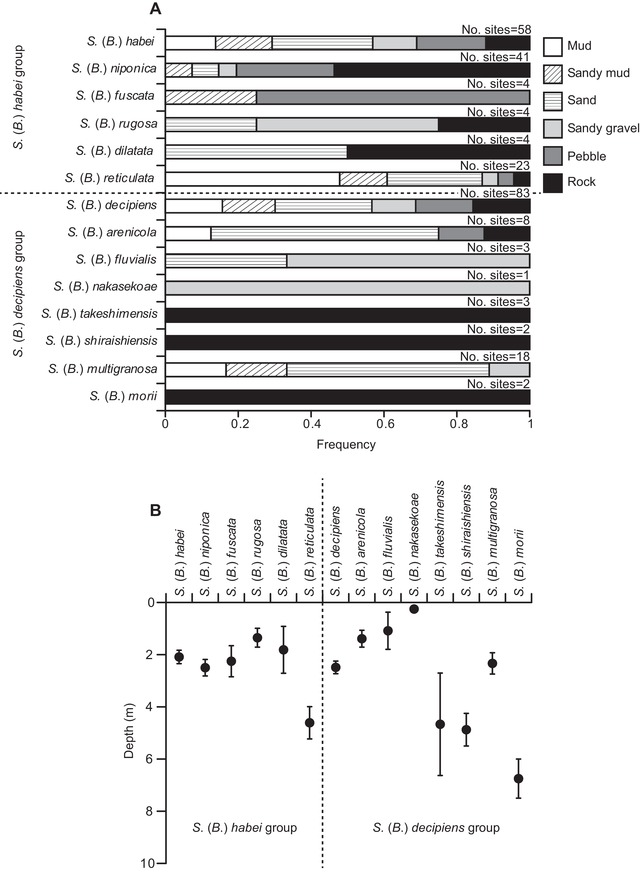
Ecological divergence in the *Biwamelania* snails. (A) The habitat types occupied by the *Biwamelania* snails. (B) The average habitat depth of the *Biwamelania* snails. Error bars indicate ± S.E.

The habitat usage pattern was significantly different among *Biwamelania* species within each group (Chi‐square test, *df* = 25, *n* = 134, *X* ^2^ = 82.0, *P* < 0.01 for the *S*. (*B*.) *habei* group; *df* = 35, *n* = 120, *X* ^2^ = 64.5, *P* < 0.01 for the *S*. (*B*.) *decipiens* group, see Fig. 5A). There were also differences in habitat depth among *Biwamelania* species within each group (GLM, *F*_5, 128_ = 5.16, *P* < 0.01 for the *S*. (*B*.) *habei* group; *F*_7, 112_ = 2.79, *P* = 0.01 for the *S*. (*B*.) *decipiens* group, see Fig. 5B). While the majority of *Biwamelania* species inhabited at the shallow coastal habitats, *S*. (*B*.) *reticulata*, *S*. (*B*.) *takeshimensis*, *S*. (*B*.) *shiraishiensis*, and *S*. (*B*.) *morii* were often found at the habitat about 4 m in depth and sometimes found at the locations deeper than 10 m.

## Discussion

Molecular phylogeny based on the ddRAD sequencing technique showed that there are two largely distinct groups in the subgenus *Biwamelania* (Fig. 2A, B). The *S*. (*B*.) *habei* group is basal and the oldest clade of *Semisulcospira* in Japan. The other clade, *S*. (*B*.) *decipiens* group, is a sister group of clade L2 of the common riverine species, *S. libertina*, which is a polyphyletic species composed of geographically and genetically well‐isolated clades (L1–L3). These two endemic species groups and three riverine clades should be taxonomically revised in future study.

Molecular phylogenies provide a framework for studying endemic radiations. Our tree rejected the hypothesis that the *Biwamelania* species flock originated from a single colonization event (Fig. 2A, B). The tree topology is most plausibly interpreted by assuming two independent colonizations of *Biwamelania* in Lake Biwa. The first colonization by the *S*. (*B*.) *habei* group occurred at the initial stage of Lake Biwa, and the second colonization by the *S*. (*B*.) *decipiens* group occurred about 1.90 million years ago (1.28–2.49 million years ago: 95% HPD; Fig. 3A) when Lake Biwa was a group of shallow swamps (Satoguchi 2012). Multiple colonizations have often been reported in other endemic radiations in ancient lakes. For example, the cichlids in Lake Malawi were colonized in the lake at least two times (Joyce et al. 2011). Further, *Tylomelania* snails in ancient lakes in Sulawesi colonized four times from surrounding rivers (von Rintelen et al. 2004). These colonization events often facilitated diversifications of lineages in ancient lakes, while not all colonizations resulted in diversification scenario (e.g., Peart et al. 2014). Matsuoka and Miura (2018) carefully inspected the adult and embryonic shells of five fossil species from the Pleistocene Katata Formation (1.2–0.4 million years ago) and found that *S*. (*B*.) *nakamurai*, *S*. (*B*.) *pseudomultigranosa*, *S*. (*B*.) *kokubuensis*, and *S*. (*B*.) *pusilla* have shell characters similar to the extant species in the *S*. (*B*.) *decipiens* group while the adult and embryonic shell of *S*. (*B*.) *spinulifera* resembles to those of the extant species in the *S*. (*B*.) *habei* group. This morphological observation is consistent with the result of molecular dating, which shows that two groups have coexisted in the lake since 1.9 million years ago (Fig. 3A). On the other hand, *S*. (*B*.) *praemultigranosa*, *S*. (*B*.) *reticulataformis* MS, *S*. (*B*.) *nojirina* MS, *S*. (*B*.) *gamoensis* MS, and *S*. (*B*.) *tagaensis* MS existed in the lake before the colonization of the *S*. (*B*.) *decipiens* group (Fig. 1C), suggesting these five old fossil species are the ancestral species in the *S*. (*B*.) *habei* group. Detailed morphological examination of these fossil species will provide an opportunity to evaluate the validity of this hypothesis.

The fossil records and the geology of the lake suggested that lake‐size change is a major factor for extinction and speciation (Fig. 1C). The latest extinction event in Lake Biwa occurred about 0.4 million years ago, when the lake basin was substantially enlarged and deepened as a result of a fault‐block movement called Rokko Movements (Matsuoka 1987). Six *Biwamelania* species were present in the fossil record before this extinction event (Matsuoka 1987; Matsuoka and Miura 2018). However, all but one species were extirpated, and *S*. (*B*.) *habei* is the only species that appears both in the past and current lake (Fig. 1C). Therefore, the fossil evidence suggests that more than 10 species of extant *Biwamelania* snails rapidly radiated following the latest expansion of the lake (Nishino and Watanabe 2000). If this fossil‐based inference is correct, we should expect to find a shallow, bushy phylogeny among *Biwamelania* species. Consistent with thisexpectation, we found that the phylogenetic relationships within the *S*. (*B*.) *habei* and *S*. (*S*.) *decipiens* groups were characterized by short branches (Fig. 2). The molecular dating analysis demonstrated that the divergence event of the *S*. (*B*.) *habei* group began about 0.35 million years ago and the *S*. (*S*.) *decipiens* group began to diverge about 0.17 million years ago (Fig. 3A), demonstrating that the radiation events in *Biwamelania* occurred following the latest increase in lake size. The net diversification rate also increased after the enlargement of the lake (Fig. 3B), further supporting the recent radiation of the *Biwamelania* snails.

The demographic reconstruction of the *Biwamelania* species demonstrated that the population of the most of *Biwamelania* snail expanded after the latest enlargement of the lake about 0.4 million years ago (Fig. 4). Similar patterns were reported in several fish populations in Lake Biwa. The demographic inferences based on mtDNA variations showed that 22 fish species or clades in Lake Biwa expanded their populations after the latest enlargement of the lake (Tabata et al. 2016). These demographic patterns suggest that the latest enlargement of the lake provided new stable habitats for faunas in Lake Biwa and resulted in the concurrent demographic increases across the diverse faunas.

New ecological niches should become available with the expansion of Lake Biwa. Adaptive radiation can take place when founders enter a new environment with a number of discrete ecological niches (Gavrilets and Losos 2009), and thus, the exploitation and specialization to new habitats is likely to be an important process in rapid diversification of the *Biwamelania* snails. Consistent with this idea, the *Biwamelania* species radiated from two ancestral species after the latest lake expansion now use a variety of habitats in Lake Biwa. In the *S*. (*B*.) *habei* group, *S*. (*B*.) *niponica*, and *S*. (*B*.) *dilatata* were often found on rocky habitats, while *S*. (*B*.) *reticulata* was found on muddy‐sandy bottoms (Fig. 5A). Similarly, in the *S*. (*B*.) *decipiens* group, *S*. (*B*.) *takeshimensis*, *S*. (*B*.) *shiraishiensis*, and *S*. (*B*.) *morii* were observed on rocky habitats, while *S*. (*B*.) *arenicola* and *S*. (*B*.) *multigranosa* were found on muddy‐sandy bottoms (Fig. 5A). Further, some *Biwamelania* snails also extend their habitat to offshore. While most of the *Biwamelania* species prefer coastal habitats, *S*. (*B*.) *reticulata*, *S*. (*B*.) *takeshimensis*, *S*. (*B*.) *shiraishiensis*, and *S*. (*B*.) *morii* are mainly distributed in deeper habitats (Fig. 5B). These habitat usage patterns can be independently evolved between two groups. The exploitation of new habitats could result in the reduction of resource competition and are suggestive of a significant role for ecological factors in rapid diversification in *Biwamelania* snails.

The studies on species radiation in the Great East African Lakes have shown that cichlids inhabit the littoral zone were allopatrically differentiated at a small geographical scale during the expansion of the lake, owing to extreme territoriality and lack of dispersal opportunities during any life stage (Sturmbauer et al. 2011). Indeed, many cichlids are narrow endemics present only in a single stretch of continuous habitat (Ribbink et al. 1983) and exhibited low level of gene flow among local populations (Genner et al. 2010). This pattern was also reported in a shallow water catfish in Lake Tanganyika (Peart et al. 2018). Similar to the case of the cichlids and catfish in African ancient lakes, the geographic distribution of nine *Biwamelania* species is confined to small regions in the lake (Watanabe and Nishino 1995, see Fig. S2). For instance, *S*. (*B*.) *fuscata*, *S*. (*B*.) *ourense*, *S*. (*B*.) *dilatata*, and *S*. (*B*.) *rugosa* have been observed in only a single or few locations in the lake. Three species, *S*. (*B*.) *takeshimensis*, *S*. (*B*.) *shiraishiensis*, and *S*. (*B*.) *morii*, inhabit only isolated islets in the lake (Fig. S2). Further, two species, *S*. (*B*.) *fluvialis* and *S*. (*B*) *nakasekoae* are distributed at only a part in Uji River, which is a major outlet of Lake Biwa (Fig. S2). *Biwamelania* species are ovoviviparous snails with no planktonic stages, and thereby, the different coasts within the lake may have been sufficient to isolate their populations. These distribution patterns suggest that, in addition to ecological factors, spatial factors can also play an important role in the radiation of the *Biwamelania* snails in the lake.

Several other factors may facilitate radiation in the *Biwamelania* species. Variation in the radula morphology of *S*. (*B*.) *decipiens*, *S*. (*B*.) *multigranosa*, and *S*. (*B*.) *reticulata* has been observed (Watanabe 1970; Prozorova and Rasshepkina 2006). The radula morphology often reflects the trophic system, such as food resource usage pattern (Hawkins et al. 1989). It suggests that the trophic specialization may also, in part, contribute to the diversification in *Biwamelania*, as it observed in the radiation of *Tylomelania* snails at ancient lakes in Sulawesi (von Rintelen et al. 2004). In addition, the *Biwamelania* species exhibited a high level of karyotype variations among species (Burch 1968; Kobayashi 1986). Reproduction between species with different karyotypes can yield hybrids that are heterozygous for chromosomal rearrangements, and these hybrids typically have reduced fertility because of error during the first meiotic division (Forejt 1996). Therefore, these karyotype variations may also account for the evolution of rapid reproductive isolation among the *Biwamelania* species.

We demonstrated that two distinct lineages of the *Biwamelania* snails were concurrently radiated during the latest expansion of Lake Biwa. Our results exhibited the potential of the *Biwamelania* snails in Lake Biwa to serve as a useful system for determining the evolutionary factors in speciation and adaptive radiation. Further development of evolutionary models for *Biwamelania* snails with ecological, paleontological, and karyotypic perspectives should provide insight into the relative importance of each evolutionary factor on radiation in *Biwamelania* snails.

### CONFLICT OF INTEREST

We have no competing interests.

LITERATURE CITED

Albertson, R.
, 
J.
Markert
, 
P.
Danley
, and 
T.
Kocher
. 1999
Phylogeny of a rapidly evolving clade: the cichlid fishes of Lake Malawi, East Africa. Proc. Natl. Acad. Sci. USA
96:5107–5110.1022042610.1073/pnas.96.9.5107PMC21824

Albrecht, C.
, 
S.
Trajanovski
, 
K.
Kuhn
, 
B.
Streit
, and 
T.
Wilke
. 2006
Rapid evolution of an ancient lake species flock: freshwater limpets (Gastropoda: Ancylidae) in the Balkan Lake Ohrid. Org. Divers. Evol.
6:294–307.

Albrecht, C.
, 
C.
Wolff
, 
P.
Glöer
, and 
T.
Wilke
. 2008
Concurrent evolution of ancient sister lakes and sister species: the freshwater gastropod genus *Radix* in lakes Ohrid and Prespa. Hydrobiologia
615:157–167.

Benton, M. J.

1995
Diversification and extinction in the history of life. Science
268:52–58.770134210.1126/science.7701342

Bouckaert, R.
, 
J.
Heled
, 
D.
Kühnert
, 
T.
Vaughan
, 
C.‐H.
Wu
, 
D.
Xie
, et al. 2014
BEAST 2: a software platform for Bayesian evolutionary analysis. PLoS Comp. Biol.
10:e1003537.10.1371/journal.pcbi.1003537PMC398517124722319

Burch, J.

1968
Cytotaxonomy of some Japanese *Semisulcospira* (Streptoneura: Pleuroceridae). J. de Conchyl.
107:3–51.

Carstens, B. C.
, and 
L. L.
Knowles
. 2007
Estimating species phylogeny from gene‐tree probabilities despite incomplete lineage sorting: an example from *Melanoplus* grasshoppers. Syst. Biol.
56:400–411.1752050410.1080/10635150701405560

Davis, G.

1969
A taxonomic study of some species of *Semisulcospira* in Japan (Mesogastropoda: Pleuroceridae). Malacologia
7:211–294.

Drummond, A. J.
, and 
A.
Rambaut
. 2007
BEAST: Bayesian evolutionary analysis by sampling trees. BMC Evol. Biol.
7:214.1799603610.1186/1471-2148-7-214PMC2247476

Eaton, D. A.

2014
PyRAD: assembly of de novo RADseq loci for phylogenetic analyses. Bioinformatics
30:1844–1849.2460398510.1093/bioinformatics/btu121

Edgar, R. C.

2004
MUSCLE: multiple sequence alignment with high accuracy and high throughput. Nucleic Acids Res. 32:1792–1797.1503414710.1093/nar/gkh340PMC390337

Forejt, J.

1996
Hybrid sterility in the mouse. Trends Genet. 12:412–417.890913810.1016/0168-9525(96)10040-8

Gadagkar, S. R.
, 
M. S.
Rosenberg
, and 
S.
Kumar
. 2005
Inferring species phylogenies from multiple genes: concatenated sequence tree versus consensus gene tree. J. Exp. Zool. B Mol. Dev. Evol.
304:64–74.1559327710.1002/jez.b.21026

Gavrilets, S.
, and 
J. B.
Losos
. 2009
Adaptive radiation: contrasting theory with data. Science
323:732–737.1919705210.1126/science.1157966

Genner, M. J.
, 
M. E.
Knight
, 
M. P.
Haesler
, and 
G. F.
Turner
. 2010
Establishment and expansion of Lake Malawi rock fish populations after a dramatic Late Pleistocene lake level rise. Mol. Ecol.
19:170–182.2000258210.1111/j.1365-294X.2009.04434.x

Glaubrecht, M.

2008
Adaptive radiation of thalassoid gastropods in Lake Tanganyika, East Africa: morphology and systematization of a paludomid species flock in an ancient lake. Zoosyst. Evol.
84:71–122.

Hawkins, S. J.
, 
D. C.
Watson
, 
A. S.
Hill
, 
S. P.
Harding
, 
M. A.
Kyriakides
, 
S.
Hutchinson
, et al. 1989
A comparison of feeding mechanisms in microphagous, herbivorous, intertidal, prosobranchs in relation to resource partitioning. J. Molluscan Stud.
55:151–165.

Heled, J.
, and 
A. J.
Drummond
. 2008
Bayesian inference of population size history from multiple loci. BMC Evol. Biol.
8:289.1894739810.1186/1471-2148-8-289PMC2636790

Hohenlohe, P. A.
, 
S. J.
Amish
, 
J. M.
Catchen
, 
F. W.
Allendorf
, and 
G.
Luikart
. 2011
Next‐generation RAD sequencing identifies thousands of SNPs for assessing hybridization between rainbow and westslope cutthroat trout. Mol. Ecol. Resour.
11:117–122.2142916810.1111/j.1755-0998.2010.02967.x

Joyce, D. A.
, 
D. H.
Lunt
, 
M. J.
Genner
, 
G. F.
Turner
, 
R.
Bills
, and 
O.
Seehausen
. 2011
Repeated colonization and hybridization in Lake Malawi cichlids. Curr. Biol.
21:R108–R109.2130027110.1016/j.cub.2010.11.029

Köhler, F.

2016
Rampant taxonomic incongruence in a mitochondrial phylogeny of *Semisulcospira* freshwater snails from Japan (Cerithioidea: Semisulcospiridae). J. Molluscan Stud.
82:268–281.

Kamiya, S.
, 
M.
Shimamoto
, and 
T.
Hashimoto
. 2011
Allozyme analysis of Japanese *Semisulcospira* species (Gastropoda: Pleuroceridae) reveals that Lake Biwa endemic species are not monophyletic. Am. Malacol. Bull.
29:23–26.

Kawabe, T.

1994
Formation of Lake Biwa Pp. 25–72
*in*
Research group for natural history of Lake Biwa
. The Natural History of Lake Biwa. Yasaka Shobo, Tokyo.

Kihira, H.
, 
M.
Matsuda
, and 
R.
Uchiyama
. 2003
Freshwater mollusks of Japan, including freshwater mollusks from Lake Biwa and Yodogawa. Pisces, Yokohama.

Kobayashi, T.

1986
Karyotypes of four species of the genus *Semisulcospira* in Japan. Venus
45:127–137.

Kocher, T. D.

2004
Adaptive evolution and explosive speciation: the cichlid fish model. Nat. Rev. Genet.
5:288–298.1513165210.1038/nrg1316

Kornfield, I.
, and 
P. F.
Smith
. 2000
African cichlid fishes: model systems for evolutionary biology. Annu. Rev. Ecol. Syst.
31:163–196.

Lee, T.
, 
H. C.
Hong
, 
J. J.
Kim
, and 
D.
O'Foighil
. 2007
Phylogenetic and taxonomic incongruence involving nuclear and mitochondrial markers in Korean populations of the freshwater snail genus *Semisulcospira* (Cerithioidea: Pleuroceridae). Mol. Phylogen. Evol.
43:386–397.10.1016/j.ympev.2007.02.01817400481

Lynch, M.

2010
Evolution of the mutation rate. Trends Genet. 26:345–352.2059460810.1016/j.tig.2010.05.003PMC2910838

Matsuoka, K.

1985
Pliocene freshwater gastropods from the Iga formation of the Kobiwako group, Mie Prefecture, central Japan. Trans. Proc. Palaeont. Soc. Japan
139:180–195.

Matsuoka, K.

1987
Malacofaunal succession in Pliocene to Pleistocene non‐marine sediments in the Omi and Ueno basins, central Japan. J. Earth Sci., Nagoya Univ.
35:23‐115.

Matsuoka, K.

2001
Fossil freshwater molluscs from the Tokai Group. Sci. Rep. Toyohashi Mus. Nat. Hist.
11:45–47.

Matsuoka, K.
, and 
O.
Miura
. 2018
Five new species of the genus *Semisulcospira* (Mollusca: Caenogastropoda: Semisulcospiridae) from the Pleistocene Katata Formation of the Kobiwako Group, Shiga Prefecture, central Japan. Bull. Mizunami Fossil Mus.
44:59–67.

Mirarab, S.
, and 
T.
Warnow
. 2015
ASTRAL‐II: coalescent‐based species tree estimation with many hundreds of taxa and thousands of genes. Bioinformatics
31:i44–i52.2607250810.1093/bioinformatics/btv234PMC4765870

Miura, O.
, 
F.
Köhler
, 
T.
Lee
, 
J.
Li
, and 
D.
O'Foighil
. 2013
Rare, divergent Korean *Semisulcospira* spp. mitochondrial haplotypes have Japanese sister lineages. J. Molluscan Stud.
79:86–89.

Miura, O.
, 
M. E.
Torchin
, 
E.
Bermingham
, 
D. K.
Jacobs
, and 
R. F.
Hechinger
. 2012
Flying shells: historical dispersal of marine snails across Central America. Proc. R. Soc. B
279:1061–1067.10.1098/rspb.2011.1599PMC326714621920976

Nishino, M.
, and 
N.
Watanabe
. 2000
Evolution and endemism in Lake Biwa, with special reference to its gastropod mollusc fauna. Adv. Ecol. Res.
31:151‐180.

Owen, R.
, 
R.
Crossley
, 
T.
Johnson
, 
D.
Tweddle
, 
I.
Kornfield
, 
S.
Davison
, et al. 1990
Major low levels of Lake Malawi and their implications for speciation rates in cichlid fishes. Proc. R. Soc. B
240:519–553.

Peart, C. R.
, 
R.
Bills
, 
M.
Wilkinson
, and 
J. J.
Day
. 2014
Nocturnal claroteine catfishes reveal dual colonisation but a single radiation in Lake Tanganyika. Mol. Phylogen. Evol.
73:119–128.10.1016/j.ympev.2014.01.01324503480

Peart, C. R.
, 
K. K.
Dasmahapatra
, and 
J. J.
Day
. 2018
Contrasting geographic structure in evolutionarily divergent Lake Tanganyika catfishes. Ecol. Evol.
8:2688–2697.2953168610.1002/ece3.3860PMC5838041

Peterson, B. K.
, 
J. N.
Weber
, 
E. H.
Kay
, 
H. S.
Fisher
, and 
H. E.
Hoekstra
. 2012
Double digest RADseq: an inexpensive method for de novo SNP discovery and genotyping in model and non‐model species. PLoS One
7:e37135.2267542310.1371/journal.pone.0037135PMC3365034

Prozorova, L. A.
, and 
A. V.
Rasshepkina
. 2006
On the radula and pallial gonoduct morphology of the gastropod *Biwamelania decipiens* and *B. multigranosa* (Cerithioidea: Pleuroceridae: Semisulcospirinae). Bull. Russian Far East Malacol. Soc.
10:130–132.

Rabosky, D. L.

2014
Automatic detection of key innovations, rate shifts, and diversity‐dependence on phylogenetic trees. PloS one
9:e89543.2458685810.1371/journal.pone.0089543PMC3935878

Rabosky, D. L.
, 
M.
Grundler
, 
C.
Anderson
, 
P.
Title
, 
J. J.
Shi
, 
J. W.
Brown
, et al. 2014
BAMM tools: an R package for the analysis of evolutionary dynamics on phylogenetic trees. Methods Ecol. Evol.
5:701–707.

Ribbink, A. J.
, 
B. A.
Marsh
, 
A. C.
Marsh
, 
A. C.
Ribbink
, and 
B. J.
Sharp
. 1983
A preliminary survey of the cichlid fishes of rocky habitats in Lake Malawi. S. Afr. J. Zool.
18:149–310.

Satoguchi, Y.

2012
Geological History of Lake Biwa Pp. 9–16
*in*
KawabeH., NishinoM., and MaehataM. (eds.). Lake Biwa: interactions between nature and people. Springer, Berlin Heidelberg.

Satoguchi, Y.
, and 
Y.
Nagahashi
. 2012
Tephrostratigraphy of the Pliocene to Middle Pleistocene series in Honshu and Kyushu islands, Japan. Island Arc
21:149–169.

Schultheiß, R.
, 
B.
Van Bocxlaer
, 
T.
Wilke
, and 
C.
Albrecht
. 2009
Old fossils–young species: evolutionary history of an endemic gastropod assemblage in Lake Malawi. Proc. R. Soc. B
276: 2837–2846.10.1098/rspb.2009.0467PMC283995819439440

Seehausen, O.

2006
African cichlid fish: a model system in adaptive radiation research. Proc. R. Soc. B
273:1987‐1998.10.1098/rspb.2006.3539PMC163548216846905

Stamatakis, A.

2014
RAxML version 8: a tool for phylogenetic analysis and post‐analysis of large phylogenies. Bioinformatics
30:1312–1313.2445162310.1093/bioinformatics/btu033PMC3998144

Strong, E. E.
, and 
F.
Köhler
. 2009
Morphological and molecular analysis of ‘*Melania’ jacqueti* Dautzenberg and Fischer, 1906: from anonymous orphan to critical basal offshoot of the Semisulcospiridae (Gastropoda: Cerithioidea). Zool. Scr.
38:483–502.

Sturmbauer, C.

1998
Explosive speciation in cichlid fishes of the African Great Lakes: a dynamic model of adaptive radiation. J. Fish Biol.
53:18‐36.

Sturmbauer, C.
, 
S.
Baric
, 
W.
Salzburger
, 
L.
Rüber
, and 
E.
Verheyen
. 2001
Lake level fluctuations synchronize genetic divergences of cichlid fishes in African lakes. Mol. Biol. Evol.
18:144–154.1115837310.1093/oxfordjournals.molbev.a003788

Sturmbauer, C.
, 
M.
Husemann
, and 
P. D.
Danley
. 2011
Explosive speciation and adaptive radiation of East African cichlid fishes Pp. 333–362. *in*
ZachosF. E. and HabelJ. C. (eds.). Biodiversity hotspots. Springer, Berlin Heidelberg.

Sturmbauer, C.
, and 
A.
Meyer
. 1992
Genetic divergence, speciation and morphological stasis in a lineage of African cichlid fishes. Nature
358:578–581.150171210.1038/358578a0

Tabata, R.
, 
R.
Kakioka
, 
K.
Tominaga
, 
T.
Komiya
, and 
K.
Watanabe
. 2016
Phylogeny and historical demography of endemic fishes in Lake Biwa: the ancient lake as a promoter of evolution and diversification of freshwater fishes in western Japan. Ecol. Evol.
6:2601–2623.2706624410.1002/ece3.2070PMC4798153

Van Bocxlaer, B.

2017
Hierarchical structure of ecological and non‐ecological processes of differentiation shaped ongoing gastropod radiation in the Malawi Basin. Proc. R. Soc. B
284:20171494.10.1098/rspb.2017.1494PMC559784228904143

Van Bocxlaer, B.
, and 
G.
Hunt
. 2013
Morphological stasis in an ongoing gastropod radiation from Lake Malawi. Proc. Natl. Acad. Sci. USA
110:13892–13897.2392461010.1073/pnas.1308588110PMC3752226

von Rintelen, T.
, 
A.
Wilson
, 
A.
Meyer
, and 
M.
Glaubrecht
. 2004
Escalation and trophic specialization drive adaptive radiation of freshwater gastropods in ancient lakes on Sulawesi, Indonesia. Proc. R. Soc. B
271:2541‐2549.10.1098/rspb.2004.2842PMC169189315615679

Watanabe, N.
, and 
M.
Nishino
. 1995
A study on taxonomy and distribution of the freshwater snail, genus *Semisulcospira* in Lake Biwa, with description of eight new species. Lake Biwa Study Monograph
6:1–36.

Watanabe, N. C.

1970
Studies on three species of *Semisulcospira* in Lake Biwa II) Comparative studies of radulae. Venus
29:93–98.

Yang, Z.

2015
The BPP program for species tree estimation and species delimitation. Curr. Zool.
61:854–865.

Associate Editor: A. Goswami

## Supporting information


**Figure S1**. Shells of representatives of the genus *Biwamelania* used in this study.
**Figure S2**. Geographical distribution of nine *Biwamelania* species with limited distribution ranges.

**Table S1**. The sampling localities of Semisulcospira spp. and the summary of RAD loci analyzed in this study.

## Data Availability

Genome DNA dataset used in this study is available at DDBJ Sequence Read Archive (accession nos. DRA004774 and DRA05712). The alignment of the concatenated DNA sequences and the ML tree are available from the TreeBASE depository (http://purl.org/phylo/treebase/phylows/study/TB2:S23499).
