# Supplementary material for: Recent lake expansion triggered the adaptive radiation of freshwater snails in the ancient Lake Biwa
Source: Evol Lett. 2018 Nov 30;3(1):43–54. doi: 10.1002/evl3.92 (PMC6369999; doi:10.1002/evl3.92)
Supplement: Supplementary file 1 — Figure S1. Shells of representatives of the genus Biwamelania used in this study. Figure S2. Geographical distribution of nine Biwamelania species with limited distribution ranges. [file EVL3-3-43-s001.docx]

Supporting figures for

**Recent lake expansion triggered the adaptive radiation of freshwater snails in the ancient Lake Biwa**

Osamu Miura, Misako Urabe, Tomohiro Nishimura, Katsuki Nakai, Satoshi Chiba

This supplement contains:

Figure S1

Figure S2

**
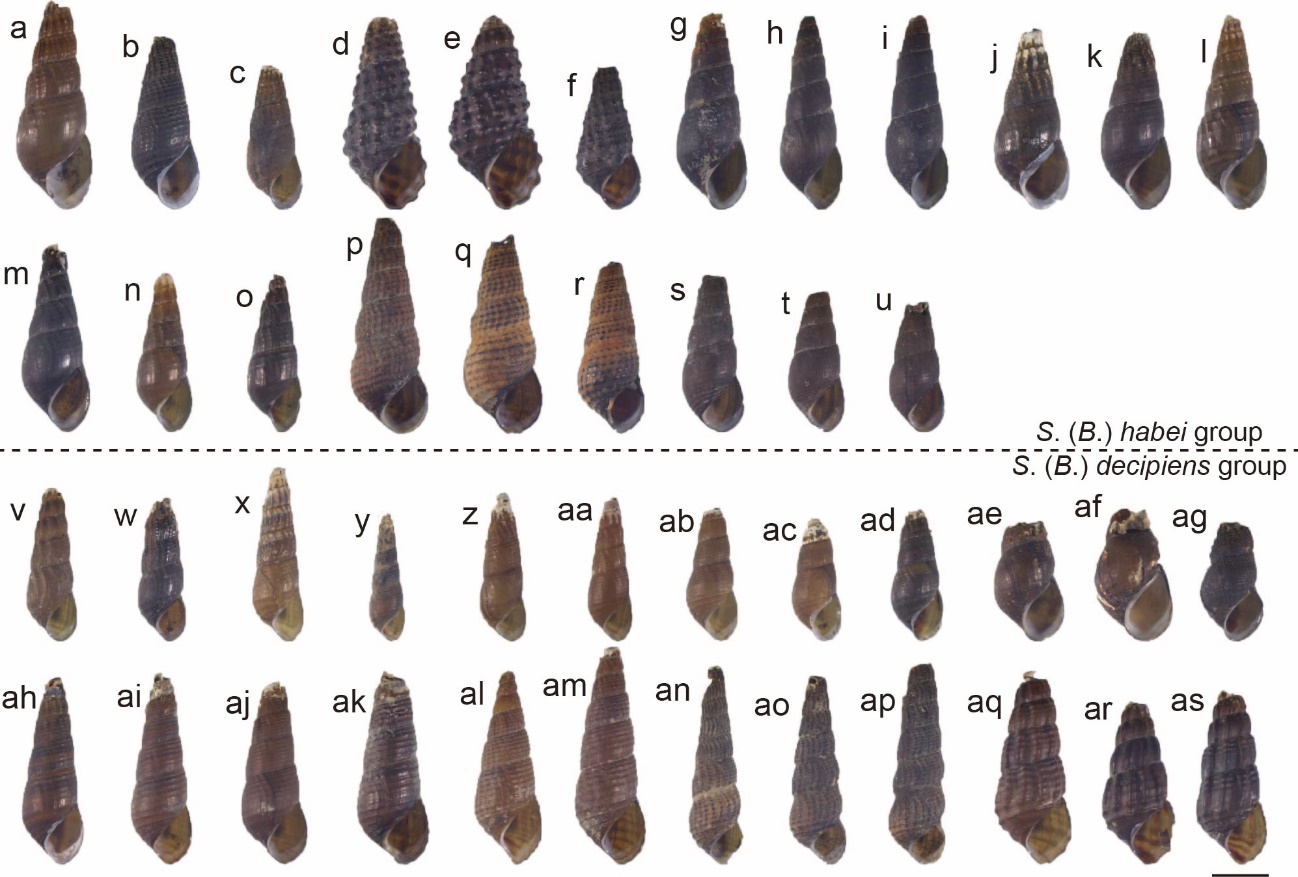
**

**Figure S1.** Shells of representatives of the genus *Biwamelania* used in this study. Specimens a-u shows the species in the *S*. (*B*.) *habei* group. a-c: *S*. (*B*.) *habei* (KUZ-Z1702, KUZ-Z1733, Z1734), d-f: *S*. (*B*.) *niponica* (KUZ-Z1689, Z1720, Z1726), g-i: *S*. (*B*.) *fuscata* (KUZ-Z1711－1713), j-l: *S*. (*B*.) *dilatata* (KUZ-Z1687, Z1695, Z1696), m-o: *S*. (*B*.) *rugosa* (KUZ-Z1717－1719), p-r: *S*. (*B*.) *reticulata* (KUZ-Z1684, Z1705, Z1706), s-u: *S*. (*B*.) *kurodai* (KUZ-Z1739－1741). Specimens v-as shows the species in the species in the *S*. (*B*.) *decipiens* group. v-x: *S*. (*B*.) *decipiens* (KUZ-Z1701, Z1716, Z1724), y-aa: *S*. (*B*.) *arenicola* (KUZ-Z1685, Z1693, Z1694), ab-ad: *S*. (*B*.) *fluvialis* (KUZ-Z1729－1731), ae-ag: *S*. (*B*.) *nakasekoae* (KUZ-Z1735－37), ah-aj: *S.* (*B*.) *takeshimensis* (KUZ-Z1690－1692), ak-am: *S*. (*B*.) *shiraishiensis* (KUZ-Z1721－1723), an-ap: *S*. (*B*.) *multigranosa* (KUZ-Z1681－1683), aq-as: *S*. (*B*.) *morii* (KUZ-Z1698－1700). The bar at the right bottom indicates 1 cm.

**
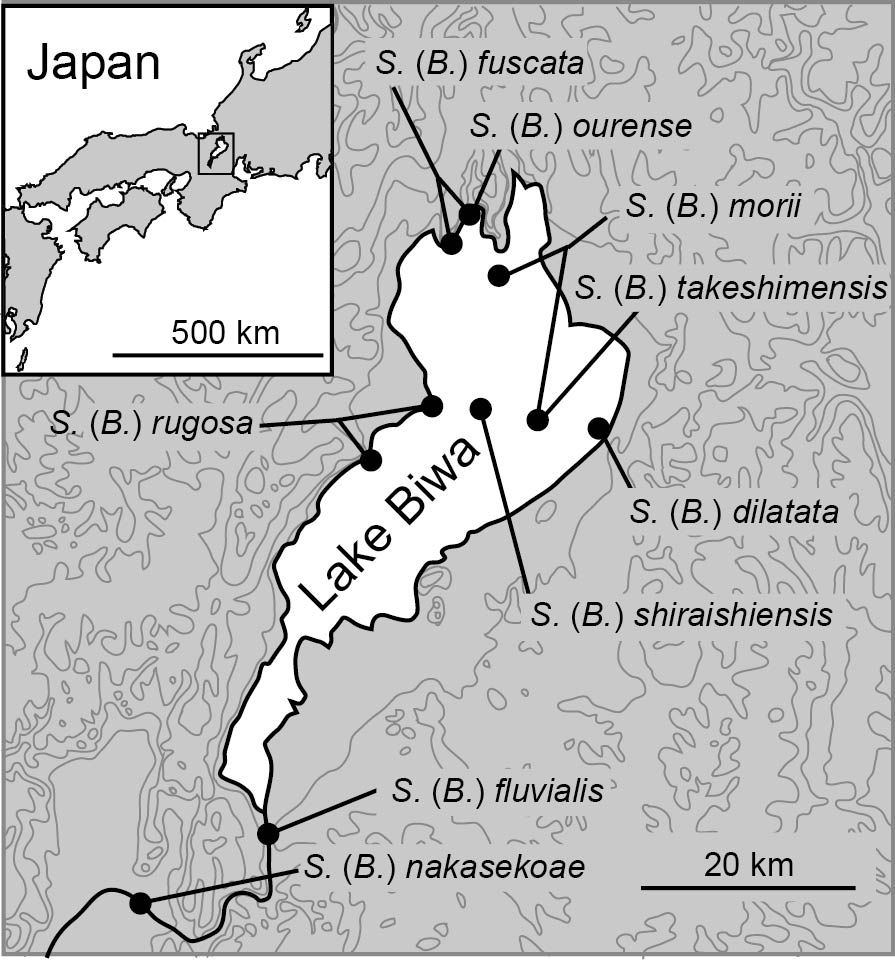
**

**Figure S2.** Geographical distribution of nine *Biwamelania* species with limited distribution ranges. The other five species (*S*. (*B*.) *habei*, *S*. (*B*.) *niponica*, *S*. (*B*.) *decipiens*, *S*. (*B*.) *arenicola*, and *S*. (*B*.) *multigranosa*) have broad distributions in Lake Biwa and are not shown on the map.
